# An overview of the placenta's role in the development of congenital diaphragmatic hernia

**DOI:** 10.3389/fped.2026.1783068

**Published:** 2026-03-10

**Authors:** Nilhan Torlak, Marc Oria, Braxton Forde, Jose L. Peiro, Emrah Aydin

**Affiliations:** 1 Department of Surgery, New York University Grossman School of Medicine, New York, NY, United States; 2 Center for Fetal and Placental Research, Division of Pediatric General and Thoracic Surgery, Cincinnati Children’s Hospital Medical Center (CCHMC), Cincinnati, OH, United States; 3 Division of Maternal Fetal Medicine, Department of Obstetrics and Gynecology, University of Cincinnati College of Medicine, Cincinnati, OH, United States; 4 Graduate School of Health Sciences, Koç University, Istanbul, Türkiye; 5 Department of Radiation Oncology, University of Cincinnati College of Medicine, Cincinnati, OH, United States; 6 Division of Maternal Fetal Medicine, Jackson Fetal Care, Department of Obstetrics, Gynecology and Reproductive Sciences, Miller School of Medicine, University of Miami, Miami, FL, United States

**Keywords:** congenital diaphragmatic hernia, fetal vascular malperfusion, placenta, retinoic acid pathway, vasculature

## Abstract

Congenital diaphragmatic hernia (CDH) is a severe congenital malformation resulting from incomplete diaphragm development, leading to abdominal organ herniation into the thoracic cavity. This disruption compromises pulmonary development, frequently resulting in lung hypoplasia and pulmonary hypertension. While the role of the placenta in congenital heart defects is well established, its involvement in congenital lung diseases, particularly CDH, remains unexplored. During the prenatal period, the placenta serves as a crucial site for nutrient and gas exchange between the mother and the fetus, and given its functional connection to fetal development, it provides a compelling avenue for investigating the pathophysiology of CDH. This review synthesizes current knowledge regarding the placental contribution to CDH, with a focus on molecular pathways, particularly the retinoic acid pathway and placental abnormalities. Evidence from both animal models and human studies suggests a complex interplay between placental function and CDH pathogenesis. Further investigation is required to elucidate the placenta's role in disease mechanisms, which may offer perspectives for future research, advances in prenatal diagnostics, and therapeutic strategies.

## 1 Introduction

### 1.1 The placenta's role in fetal organ function and development

Congenital malformations develop due to the disruption of organ morphogenesis during fetal development, driven by genetic and environmental factors. Despite extensive research, the etiopathogenesis of many fetal defects remains largely unknown. Congenital lung anomalies, notably congenital diaphragmatic hernia (CDH), present significant challenges in pediatric and perinatal medicine. CDH is characterized by incomplete formation of diaphragmatic musculature, followed by herniation of the abdominal organs into the thoracic cavity, leading to significant complications, including lung hypoplasia and pulmonary hypertension (1).

**Figure 1:**
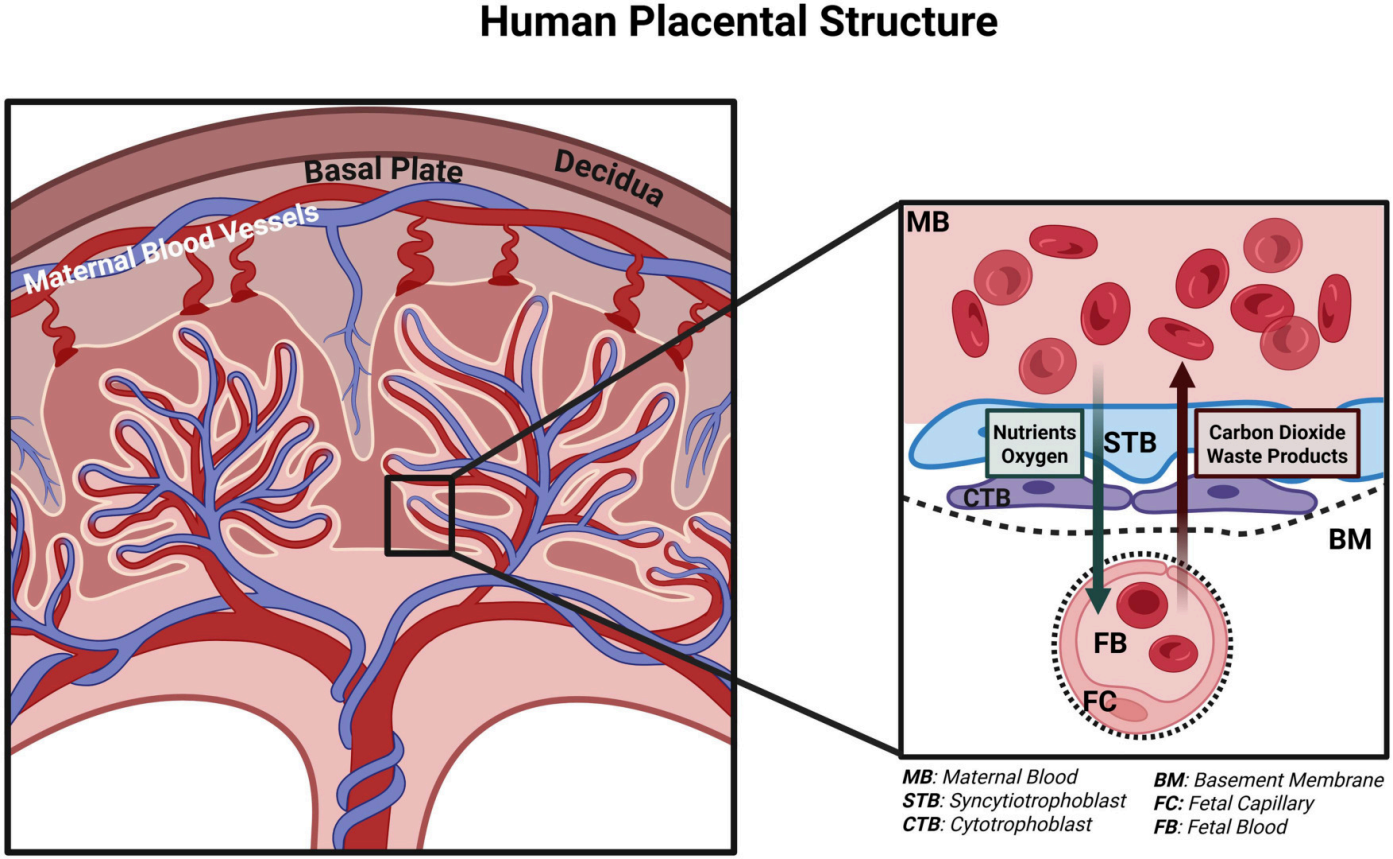
Human placental structure describing the nutrient and gas exchange between maternal and fetal units. Intervillious space containing the maternal blood (MB) is in direct contact with syncytiotrophoblasts (STBs) for nutrient and gas exchange, with a layer of cytotrophoblasts (CTBs) and basement membrane (BM) between the fetal capillaries (FC) containing fetal blood (FB). Created in BioRender. Torlak N. (2026) https://BioRender.com/aeowwx0.

Historically, the role of the placenta in fetal lung development has remained underexplored. However, contemporary research has begun to unravel the placenta's multifaceted contributions, particularly in the context of CDH. During prenatal development, the placenta serves as the primary site for nutrient and gas exchange between the mother and the fetus, ensuring proper fetal growth (Figure 1). In more than 99% of cases, the placenta and the fetus share the same genetic information. Given the placenta's genetic and functional connection to fetal development, it should be considered for the investigation of underlying pathophysiological aspects of congenital diseases.

There is robust biological evidence for the placenta's role in fetal pathologies. In congenital heart diseases (CHD), the most common congenital anomaly, single-cell sequencing of fetal and placental structures has identified parallel abnormalities in developmental pathways. This has led to placental maldevelopment, resulting in embryonic malperfusion, leading to CHD. A feedback loop is then established where placental dysfunction contributes to abnormal fetal heart structure, further worsening fetoplacental malperfusion. This cycle exacerbates placental structure and functional deficiencies (2–4).

Regarding the placenta's role in neonatal lung function, studies have demonstrated an association between placental abnormalities and bronchopulmonary dysplasia (BPD), the most common severe respiratory morbidity in neonates. Research involving premature infants with BPD and pulmonary hypertension (PHT) has shown that placental villous vascularity is significantly reduced compared to the non-PHT control group (5). Another study investigating the correlation between chorionic plate vascularization and the risk of BPD found a strong association between perforating chorionic vessel density (PCVD) and the severity of subsequent BPD development (6). While the placenta's impact on neonatal lung function is increasingly recognized, its specific role in congenital lung diseases such as CDH remains poorly understood.

This review focuses on the placenta's critical role in the development of fetal diseases, specifically in CDH, by synthesizing findings from experimental models and human CDH cases. By investigating the placenta's involvement in CDH pathophysiology, we aim to reveal new areas for research by demonstrating placental disruptions as a contributing factor in CDH, and to identify diagnostic markers and therapeutic interventions.

### 1.2 Placenta in congenital diaphragmatic hernia

Congenital diaphragmatic hernia (CDH) is a developmental anomaly with an incidence rate of 1 in 2,500–5,000 births, characterized by a defect in the diaphragm that allows abdominal organs to herniate into the thoracic cavity. This displacement compromises pulmonary development due to compression and competition for the intrathoracic space. This condition is primarily defined by varying degrees of pulmonary hypoplasia (PH) and pulmonary hypertension (PHT). Clinically, the most common type of CDH, posterolateral CDH, also known as Bochdalek hernia, accounts for 90%–95% of human CDH cases, and while most of these involve a left-sided diaphragmatic defect, it can be right-sided as well (7–10). Additionally, Morgagni hernia (anteromedial hernia) constitutes 2%–5% of CDH cases, while the defect can rarely be central or bilateral as well (8, 11, 12). The side of the diaphragmatic defect is crucial due to its effect on prognosis, abdominal organ herniation, survival, and possibly underlying molecular aspects (13).

While initially attributed to mechanical compression by herniated organs, CDH is now understood to involve complex molecular pathways affecting lung development, owing to the “dual-hit hypothesis” proposed in the 2000s, explaining the embryogenesis of CDH through the nitrofen (2, 4-dichlorophenyl-p-nitrophenyl ether) induced model, which paved the way for the following studies on CDH pathogenesis and underlying molecular pathways related to both PH and PHT (14). Several examples for these pathways can be but are not limited to, retinoic acid, sonic hedgehog (SHH), and fibroblast growth factor (FGF) signaling pathways for PH and vascular endothelial growth factor (VEGF), endothelin 1 (ET-1) and nitric oxide (NO) signaling pathways for PHT (15, 16). The features caused by dysregulation of these pathways include impaired gas exchange, reduced alveolar airspace, thickened alveolar walls, abnormal vascular beds, and excessive smooth muscle cell proliferation (15–18). Altered pulmonary arterial branching and architecture contributed to the generation of significant distal vasculature resistance and PHT, which can manifest in cardiac right ventricular structural and functional abnormalities (19). In addition, in left-sided CDH, compression can lead to ventricular hypoplasia, or more severely hypoplastic left heart syndrome (HLHS) involving underdevelopment of aortic valve and aorta with left ventricle (20, 21). These findings highlight CDH as a systemic vascular disorder affecting multiple organs to varying degrees.

Recent human studies provided new insights into CDH's impact on fetoplacental vasculature. A retrospective analysis comparing placentas from CDH pregnancies to those with umbilical cord pathology but without CDH identified features of large vessel and remote distal villous fetal vascular malperfusion (FVM). These findings suggested that CDH-associated mass effects impair placental blood flow, and the reported FVM was earlier than that of umbilical cord compromise (22). Another study investigating prenatal brain maturation in CDH cases reported high incidences of placental chronic inflammation (67%, chronic villitis or chronic deciduitis with plasma cells), FVM (59%, fetal vascular thrombus, multiple vascular thrombi or high-grade FVM), and umbilical cord abnormalities (54%, hypercoiled umbilical cord or insertion abnormality) (23). Through these findings, they concluded that the delayed fetal brain maturation in CDH cases can be associated with the identified placental pathologies.

**Figure 2:**
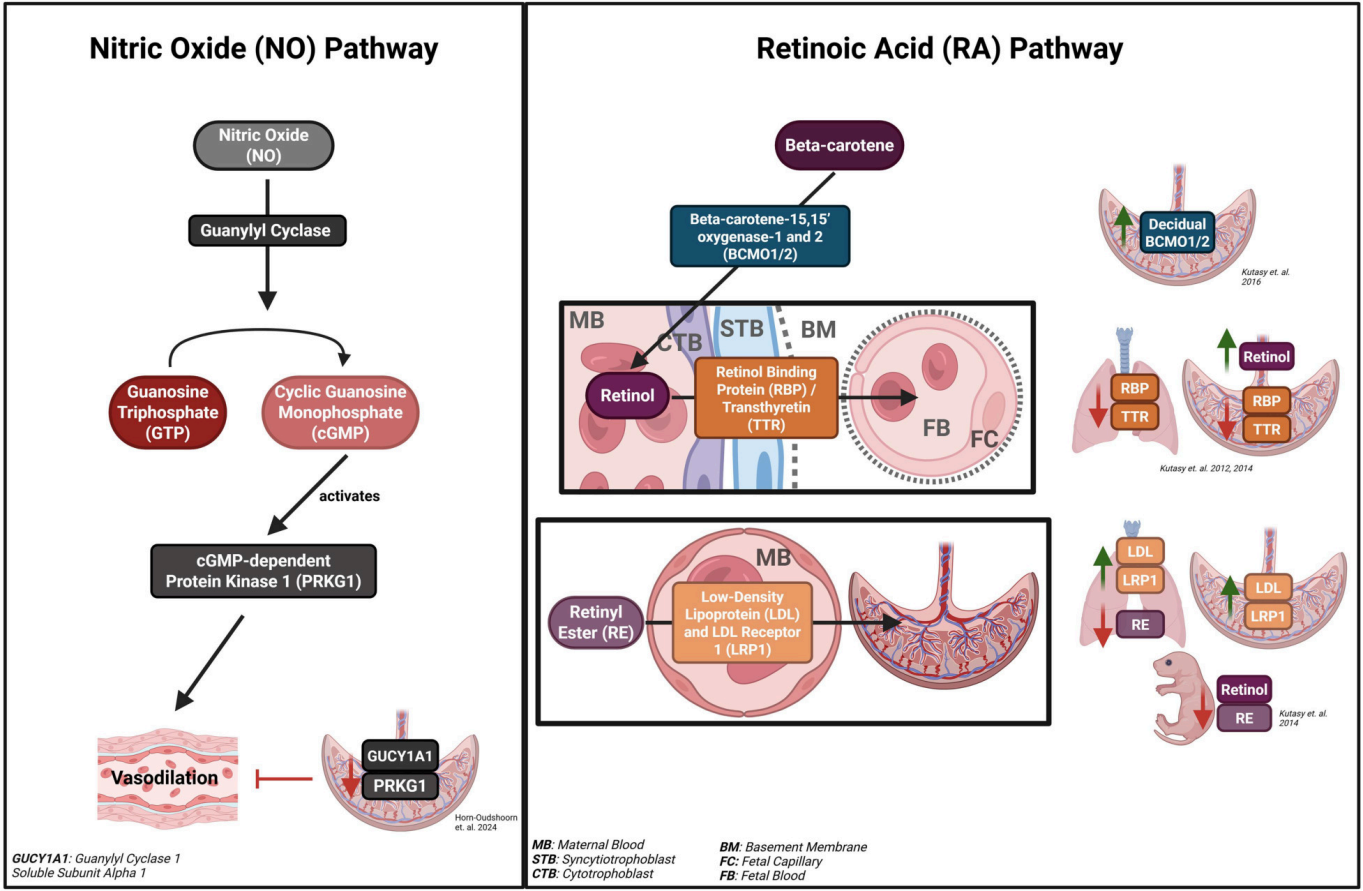
Nitric oxide (NO) and retinoic acid (RA) pathways and their components investigated in the placenta in the context of congenital diaphragmatic hernia (CDH). Created in BioRender. Torlak N. (2026) https://BioRender.com/duhiro6.

Moreover, a recent study analyzing fetoplacental artery reactivity isolated from the placentas of infants with CDH found that these arteries exhibited greater vasoconstriction in response to the thromboxane A2 agonist, a potent vasoconstrictor, and reduced dilation in response to vasodilators such as bradykinin and nitric oxide-donor sodium nitroprusside (24). Notably, key nitric oxide (NO) pathway components, including guanylyl cyclase 1 soluble subunit alpha 1 (GUCY1A1) and cyclic guanosine monophosphate (cGMP) dependent protein kinase 1 (PRKG1), exhibited decreased mRNA expressions in CDH placentas compared to healthy placentas, indicating disrupted vasodilation (24). The NO pathway is particularly relevant in CDH due to its role in pulmonary vasodilation via guanylyl cyclase activation and cGMP production (Figure 2) (25). This research group suggests that the disrupted response to induced NO (iNO) during postnatal treatment for CDH-induced PHT may be explained by the impaired placental NO pathway described via their findings (24, 26). These findings indicate that placental vascular alterations in CDH may mirror those observed in pulmonary vasculature, potentially influencing responses to pulmonary vasodilatory therapies.

In addition to the established similar vasoreactive responses, abnormal pulmonary vascular development, which is a key determinant of CDH severity, may be modulated by placental factors such as vascular endothelial growth factor A (VEGFA) and placental growth factor (PLGF). It is crucial to note that PLGF is primarily sourced by the placenta itself, and with soluble fms-like tyrosine kinase (sFLT-1) serves as a marker for preeclampsia and placental dysfunction (27, 28). With VEGFA, they are members of the VEGF family involved in angiogenesis, and studies have reported elevated plasma VEGFA and reduced PLGF levels in CDH cases; therefore, they established a direct correlation of these changes with the severity of pulmonary hypertension in CDH (29, 30). The limited information on molecules involved in pulmonary and placental vasculature provides evidence on how placental and pulmonary vasculature can show similarities and how placental factors can be utilized to identify CDH severity.

While clinical studies identified abnormalities in the fetoplacental vasculature in CDH cases, rodent models, specifically the nitrofen-induced CDH model, investigated only the placental retinoic acid pathway's involvement in CDH, highlighting the tremendous gap in placental research in CDH. Retinol, an essential nutrient that the fetus cannot synthesize, is transported from the placental retinol (31). Studies demonstrated impaired retinol transport due to reduced trophoblastic expression of retinol-binding proteins (RBPs) and transthyretin (TTR), with increased placental retinol accumulation, and decreased trophoblastic and fetal pulmonary expression of retinol carriers (Figure 2) (31, 32). However, the existence of alternative pathways was evidenced by healthy offspring from RBP-deficient mice (33).

Another mechanism of disrupted retinol transfer involves maternal dietary retinyl ester (RE) bound to low-density lipoprotein (LDL), mediated by LDL receptor 1 (LRP1). Placental and pulmonary expression levels of these proteins were observed to be increased, along with reduced fetal retinol, increased fetal serum RE, and diminished pulmonary RE levels in nitrofen-induced CDH (Figure 2) (34). This upregulation suggests an adaptive response to impaired retinol transport and pulmonary morphogenesis.

Beta-carotene, the primary dietary retinol source, is enzymatically cleaved into retinol by beta-carotene-15,15' oxygenase-1 and 2 (BCMO1 and 2). Supplementation with beta-carotene in vitamin A-deficient lecithin retinol acyltransferase (LRAT) and RBP knockout mice increased placental BCMO1/2 expression (35). In nitrofen-induced CDH rats, elevated maternal BCMO1/2 expression and RBP levels suggest that decidual RBP expression and retinol synthesis are preserved despite nitrofen's impairment of trophoblastic RBP expression (Figure 2) (36).

Further supporting these findings, maternal administration of retinoic acid in nitrofen-exposed pregnancies enhanced trophoblastic RBP-dependent retinol transport and upregulated genes involved in the retinol signaling pathway. This intervention improved body and lung weight, and the number of alveoli in rat fetuses (37). Additionally, during late lung morphogenesis, trophoblast atrophy and apoptosis were observed in nitrofen-exposed placentas, suggesting placental dysfunction in CDH (31, 32). There were also attributions to over-activation of neutrophil gelatinase-associated lipocalin (NGAL), a mediator of oxidative stress which mediates innate immunity, inducing trophoblastic apoptosis (38, 39). Further investigation into other molecular pathways regulating fetal and placental development, as well as placental-derived signaling molecules, is crucial to fully understand the interplay between the placenta and CDH.

## 2 Discussion

In this review, we aimed to highlight the potential role of the placenta in CDH by synthesizing existing literature from established rodent models and human cases. However, the literature focusing on placental abnormalities in CDH remains limited. The primary area of interest regarding their potential relationship centered on the retinoic acid pathway, which is critical given the absence of retinol *de novo* synthesis by the developing fetus. Recently, the area of interest has shifted to placental vasculature in human cases of CDH, yielding new insights into this crucial topic.

Studies in nitrofen-induced CDH rats have demonstrated disturbed retinol transfer, as evidenced by altered expression of retinol carriers, resulting in increased placental retinol levels. This disruption compromises the passage of retinol from the placenta to the fetal circulation, a process essential for lung morphogenesis (31, 32). Nonetheless, the retinoic acid pathway continued through an alternative route via RE-LDL-LRP1 signaling (34). This suggests that although nitrofen disrupts the primary maternal-fetal retinol transfer route, the fetal lungs attempt to compensate for impaired retinol transport. When the focus shifted to the maternal enzymes responsible for cleaving the primary dietary retinol source, beta-carotene, nitrofen was shown not to interfere with decidual expression of RBPs and retinol levels. This discrepancy underscores the complexity of nitrofen-induced disruptions, despite its status as a well-established rodent model that closely resembles human CDH pathology (36, 40).

Efforts to rescue the disrupted retinoic acid pathway have included antenatal retinol treatment in fetal rats with nitrofen-induced CDH. This intervention resulted in elevated trophoblastic expression of RBP and increased activation of retinol signaling pathway components, leading to higher fetal serum and pulmonary retinol levels. Ultimately, this treatment successfully mitigated pulmonary hypoplasia in CDH, reinforcing the critical role of the retinoic acid pathway in CDH pathogenesis and highlighting the placenta's involvement in this process (37). However, the unknowns in retinoic acid metabolism in human CDH and vitamin A's teratogenic potential making it not feasible as a treatment approach should be noted. Even though, when all the research regarding the placental retinoic acid pathway in CDH is taken into consideration, these findings reiterate the involvement and importance of the pathway in pulmonary development, highlighting how the placenta may be an active contributor to the pathophysiology of CDH.

Beyond disruptions in retinol transfer, placental abnormalities in CDH extend to structural and cellular dysregulation. Notably, nitrofen-induced CDH placentas exhibit increased trophoblastic atrophy and apoptosis, with apoptosis particularly driven by the overactivation of neutrophil gelatinase-associated lipocalin (NGAL). These findings indicate that placental dysregulation in CDH is not limited to retinol metabolism but encompasses broader pathological mechanisms (31, 32, 38). This also highlights the multifactorial nature of CDH, which requires further focus in future research concerning the placenta's involvement, potentially starting with the molecular pathways known to be involved in CDH development.

Although studies on human CDH placentas remain scarce, existing research primarily focuses on placental vasculature. The findings on FVM in CDH placentas suggest a disruption of fetal blood outflow due to the mass effect of CDH, providing a crucial connection between CDH and placental pathologies (22). While further research offered additional confirmation of FVM in CDH, it also identified associated umbilical cord abnormalities and chronic placental inflammation (23). The evidence clearly reiterates how placental disruptions can be present in human CDH cases, which may be investigated for their relation to the aspects of disrupted pulmonary development. Chronic placental inflammation in CDH cases should specifically be underlined due to its potential in worsening the outcome for CDH cases, since its known association with fetal growth restriction, preterm birth, and even pregnancy loss in cases of chronic placental inflammatory pathologies such as chronic villitis and deciduitis (41–43). Further investigation into this placental inflammation in CDH is necessary to identify the pathways responsible for this inflammatory immune response and it is also crucial to focus on due to the potential role of inflammation in interrupting lung development thereby causing pulmonary hypoplasia in CDH (44). Therefore, future research is needed to see if these two inflammatory events are sharing any pathways. Additionally, investigations using vasodilators have revealed similar alterations in placental vasculature to those of pulmonary vasculature in CDH cases, further supporting the presence of placental dysregulation in CDH (24). This study provided more evidence for our understanding of a possible connection between the placenta and CDH.

Collectively, the findings from human placental studies indicate a fetoplacental vascular phenotype in CDH that extends beyond isolated pulmonary pathology. The presence of FVM, reduced NO-mediated vasodilatory capacity, and an angiogenic imbalance characterized by elevated VEGFA and diminished PLGF altogether suggest a pattern of placental vascular dysfunction. These alterations can be interpreted within a standard pathophysiological sequence in which mechanical compression and altered fetal hemodynamics contribute to impaired placental perfusion, which in turn may further worsen pulmonary vascular abnormalities. Taken together, these data support the idea that CDH is not just a condition limited to the thoracic cavity, but rather a more comprehensive disturbance of fetoplacental vascular regulation.

These placental findings carry significant clinical implications for the prenatal and postnatal management of CDH. The variable and often limited therapeutic response to inhaled NO in affected neonates may be explained by impairment of nitric oxide signaling within the placenta. As a potential biomarker for prenatal risk assessment, the observed reduction in circulating PLGF may be extremely valuable, particularly in identifying fetuses with a more severe vascular phenotype. Evidence of fetal vascular malperfusion also suggests that altered fetoplacental hemodynamics could coexist with growth restrictions, providing new opportunities for prenatal monitoring. Furthermore, these vascular disturbances support the possibility that a subset of pulmonary hypertension observed in CDH may arise independently of the degree of lung compression, highlighting the need to evaluate contributions of the placenta to the disease severity and treatment response.

In conclusion, this review highlights three key insights that, combined together, reshape the understanding of CDH. First, the placenta is shown to be an active participant in CDH rather than a passive background organ, with evidence from both animal models and human studies. Second, evidence from the presence of fetal vascular malperfusion, impaired NO-mediated vasodilation, and altered VEGFA and PLGF profiles suggests that CDH is associated with a distinct fetoplacental vascular phenotype that parallels pulmonary vascular pathology. Third, the identification of this shared placental and pulmonary vascular disruption offers a conceptual framework that may support future treatment approaches and diagnostic biomarkers aimed at enhancing prenatal risk assessment and postnatal management in CDH.
